# Re-Examination of 30-Day Survival and Relapse Rates in Patients with Thrombotic Thrombocytopenic Purpura-Hemolytic Uremic Syndrome

**DOI:** 10.1371/journal.pone.0127744

**Published:** 2015-05-22

**Authors:** Cassiana E. Bittencourt, Jennifer P. Ha, Robert W. Maitta

**Affiliations:** 1 Department of Pathology, University Hospitals Case Medical Center, Cleveland, Ohio, United States of America; 2 Case Western Reserve University School of Medicine, Cleveland, Ohio, United States of America; Mario Negri Institute for Pharmacological Research and Azienda Ospedaliera Ospedali Riuniti di Bergamo, ITALY

## Abstract

**Background and Objectives:**

Thrombotic thrombocytopenic purpura (TTP) and hemolytic uremic syndrome (HUS) are characterized by microangiopathic hemolytic anemia and thrombocytopenia. Interestingly, markedly different survival rates have been reported despite increases in survivability. We studied TTP-HUS 30-day mortality and relapse rates of patients who received TPE at our institution and compared them to published data.

**Patients and Methods:**

Retrospective study analyzed 30-day mortality and relapse rates attributed to TTP-HUS from 01/01/2008 to 12/31/2012 and compared them to comparable literature reporting mortality and survival. Studies describing other etiologies for TPE and different mortality time interval were excluded.

**Results:**

Fifty-nine patients were analyzed and all were initially treated with TPE and corticosteroids. Eleven patients were classified as not having TTP-HUS due to testing or clinical reassessment which ruled in other etiologies, and 18/59 patients had ADAMTS13 activity <10%. Of remaining patients, 36/48 (75%) were diagnosed as idiopathic and 12/48 (25%) as secondary TTP-HUS. Patients received a mean of 12 TPEs (range 1-42); 42/48 (87.5%) patients had ADAMTS13 activity measured; complete response obtained in 39/48 (81.2%) patients (platelet count >100 x 10^9^/L); partial response in 4/48 (8%); and 5/48 (10.4%) did not have increases in platelet counts (2/5 of these patients died within the study period). Forty percent of patients obtained platelet counts >150 x 10^9^/L. Overall 30-day mortality for our patient cohort was 6.7% (4/59). Comparison of our mortality rate to combined data of five published studies of 16% (92/571) showed a significant difference, *p* = 0.04. Our relapse rate was 18.6% (11/59) similar to previous reports.

**Conclusions:**

Wide differences in mortality may be due to grouping of two distinct pathologic entities under TTP-HUS; and presence of confounding factors in the patient populations under study such as co-morbidities, promptness of TPE initiation, delay in diagnosis and therapeutic practice.

## Introduction

Thrombotic thrombocytopenic purpura (TTP) and hemolytic uremic syndrome (HUS) are rare diseases characterized by microangiopathic hemolytic anemia (MAHA) and thrombocytopenia. HUS is characterized by three clinical signs: kidney failure, hemolytic anemia, and thrombocytopenia while TTP has been defined by a diagnostic pentad of thrombocytopenia, hemolytic anemia, fever, neurologic changes and renal compromise [1]. However, the pentad associated with TTP is today mostly academic since most patients do not present with all symptoms at once [2, 3].

At the center of the pathology initial reports listed deficiency in the von Willebrand Factor (vWF) metalloprotease ADAMTS13 as a marker of disease and as a potential factor that may aid in the differentiation of TTP from HUS [4, 5]. However, even though ADAMTS13 differences were originally described, not all patients presenting with the clinical diagnosis of TTP have a deficiency in this metalloprotease leading to the suggestion that it lacks importance in the diagnostic workup of these disease entities [6], and as predictor of clinical response to therapy which should be based mostly on clinical criteria [7].

Ever since the results of the clinical trial by the Canadian Apheresis Study Group showed the benefits of therapeutic plasma exchange (TPE) vs. simple high volume plasma infusion, the survival of TTP patients has improved significantly from the high mortality seen a quarter of a century ago [8], and has led to a seven fold improvement in outcomes including those TTP patients with severe renal compromise [9, 10]. In another study evaluating the efficacy of TPE vs. plasma infusion, the former was shown to be more beneficial in patients receiving disease-relief by plasma infusion but who did not respond well to the increased volume leading to the use of TPE in this subgroup [11].

Complications can occur in response to TPE, and the disease can still be fatal and patients can relapse despite aggressive therapy. However, reports for the last two decades have shown markedly differing survival and relapse rates during the critical treatment period with TPE. In the present study we determined the 30-day mortality rate and relapse of TTP-HUS patients at a large tertiary academic medical center and compared these to published literature reporting similar data.

## Materials and Methods

### Study design and data collection

All patient information was anonymized and de-identified prior to analysis. This retrospective study evaluated the 30-day survival and relapse rates in patients with suspected TTP-HUS at University Hospitals Case Medical Center (UHCMC), a tertiary academic medical center, from January 1^st^ 2008 to December 31^st^ 2012. All individuals given a presumptive diagnosis of TTP-HUS by the hematology clinical service, referred to the Apheresis Center (AC) at UHCMC, and who were transferred to be treated with TPE by the AC were included in the study. None of the patients received TPE at transferring institutions. Patients who had undergone apheresis for other clinical indications were excluded. The inpatient and outpatient records of all patients were identified through AC records and the hospital’s electronic medical record.

Two reviewers using standardized forms that we developed, independently collected and verified the accuracy of the data gathered which included: demographic information, duration of TPE treatment, number of TPE procedures, volume replaced, complete blood count (platelet count, hemoglobin/ hematocrit), creatinine at presentation and end of therapy, ADAMTS13 activity, neurological symptoms and/ or fever, 30 day-mortality and relapse, and presence of other co-morbidities. In order to ascertain and compare disease severity, a Clinical Severity Score (CSS) that has been previously described was used [12]. Briefly, the four parameters used in clinically scoring patients were: neurologic symptoms, renal insufficiency, platelet counts and hemoglobin concentration, if available at the time of presentation. Score ranged from 0 to 8 points. Clinical diagnostic criteria followed recommendations previously outlined [13].

Patients were grouped into two categories: idiopathic if they had no other illness that is recognized to be associated with the symptoms at presentation; or secondary TTP-HUS if the patients had an identified trigger leading to the symptoms (e.g. stem cell transplantation, pregnancy/ postpartum, drugs, infections, autoimmune diseases, or malignancy-associated). The study was approved by the Institutional Review Board of UHCMC, Cleveland, OH.

### Patient outcome definitions

Remission, response and relapse were defined as previously described [14, 15]. Briefly, remission was defined as an increase in platelet counts within a 30-day period after completion of TPE. Complete response was defined as a platelet count greater than 100 x 10^9^/L for two consecutive days (used for data comparison since three of four studies found in literature review use this platelet count to define recovery [12, 14, 16]); partial response as a platelet count in the 50–100 x10^9^/L range; and no response as platelet counts below 50 x10^9^/L. Survival was defined as achievement of remission. Mortality from TTP was defined as occurring within 30-days of stopping TPE. Relapse was defined as a decrease in platelet count below 100x10^9^/L, consistent with TTP after achievement of remission. All patients received corticosteroids at different times during their clinical presentations.

### Therapeutic Plasma Exchanges

All procedures were performed as previously described [17]. Briefly, all patients were initiated on daily TPE as soon as the presumed diagnosis of TTP-HUS was clinically made. TPE was performed daily with 1–1.5 plasma volume exchange using the COBE Spectra Apheresis System (Terumo BCT, Lakewood, CO). When indicated, patients were transfused to a hematocrit of ≥ 23% to increase the efficiency of TPE. Pre- and post-TPE fibrinogen, pro-thrombin time, partial thrombin time, ionized calcium and daily CBC were obtained. Patients were pre-medicated with 650 mg acetaminophen and 25 mg of diphenhydramine prior to TPE. All procedures used ABO-type-specific fresh-frozen plasma as replacement fluid and citrate dextrose-A solution as anticoagulation.

### ADAMTS13 activity

Forty one out of 48 (85.4%) patients had a sample collected for baseline ADAMTS13 activity prior to initiation of TPE but activity results were not available at the time of TPE initiation. ADAMTS13 activity assay was performed at the Blood Center of Wisconsin (BCW) (Milwaukee, WI) by the fluorescence resonance energy transfer (FRET) assay for ADAMTS13 activity as previously described [18]. When appropriate, metalloprotease inhibitor level was also measured by BCW.

### Literature search and study selection

**Fig 1:**
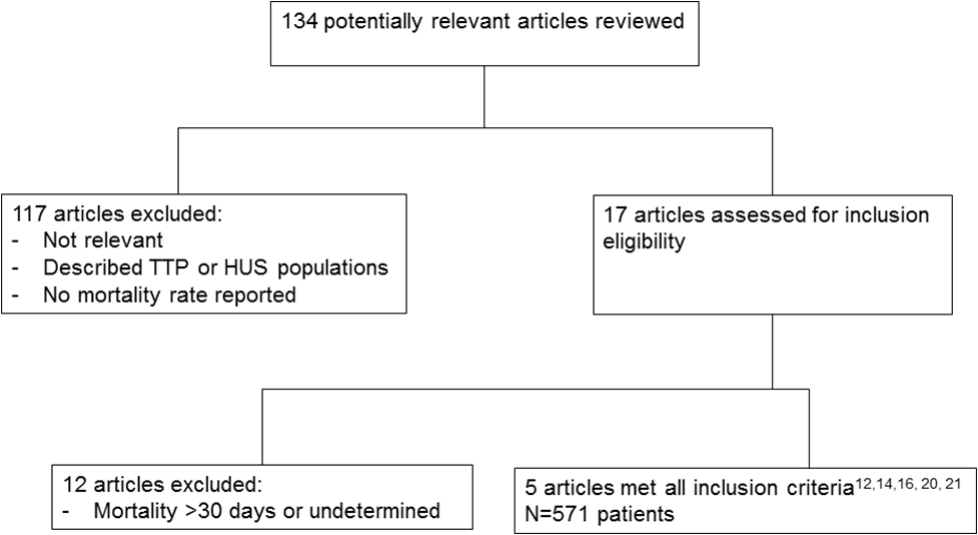
Study selection dendrogram used for data comparison. All five studies reported 30-day mortality and relapse in their study cohorts.

A comprehensive literature search strategy was conducted to identify potential articles published in English on PUBMED from 1970 to 2013. Following search keyword combinations were used: TTP-HUS, mortality/outcome, and relapse. Our search algorithm is shown in Fig 1. Selected articles were examined by two reviewers independently. Inclusion criteria were: 1) Patients with initial diagnosis of TTP-HUS, 2) 30-days mortality rate, and 3) relapse rate reported.

### Statistical Analysis

All statistics were performed using Prism 6 (GraphPad Software Inc., La Jolla, CA). Since the number of studies we found in the literature review was small and we did not have all of their raw numerical data, we were unable to perform a meta-analysis so we limited our analysis to comparisons of their results to ours. Mortality and relapse rates comparisons with similar combined data from five relevant articles were performed using a two-tailed Fisher test with a *p*< 0.05 set for significance.

## Results

### UHCMC patients

A total of 59 patients were analyzed; 11 patients had TPE discontinued as requested by the clinical team due to other diagnoses that explained these patients’ presentations. These patients were eventually diagnosed with disseminated intravascular coagulation, acute myeloid leukemia with marrow involvement, scleroderma, idiopathic thrombocytopenic purpura, malignant hypertension and sepsis/ bacteremia. Patients responded to treatment modalities which addressed each of these diagnoses after discontinuation of TPE.

The remaining 48 patients were diagnosed with TTP-HUS and received daily TPEs. The demographic characteristics of these patients at diagnosis are summarized in Table 1. Idiopathic TTP-HUS was diagnosed in 36 patients (75%) and secondary causes in 12 patients (25%). Thirty-three (69%) of patients were female, mean age of 49 (range 16–85); while 15 (31%) patients were males with mean age of 47.5 (range 28–76). There were a number of co-morbidities in our patient cohort which were as follows: two patients were pregnant/ postpartum (4%); 3 patients had a drug-associated presentation (6%); 2 patients had autoimmune disease (4%); 1 patient had a systemic infection (2%); 4 patients had malignancy/ metastasis (8%); and 1 patient had received a renal allograft (2%). Thirty-day mortality among TTP-HUS patients was 1/48 patients (2%). However, overall mortality for our patient cohort was 4/59 (6.7%) since 3 additional patients died of causes unrelated to TTP-HUS: one of these patients died due to complications of engraftment failure and graft vs. host disease (outside 30 day study period); a second died of multi-organ failure due to sepsis and bacteremia in the setting of disseminated terminal Burkitt’s lymphoma complicated by renal allograft rejection; and a third died of heart failure due to worsening ejection fraction and heart disease. Only one of the four patients who died in our cohort had an autopsy with findings that were consistent with a TTP presentation [19] and had ADAMTS13 deficiency (<5%). One male patient was treated with two cycles of TPE 3 months apart due to relapse and a second male was treated three times with TPE within a year from initial diagnosis (data not shown).

**Table 1:** UHCMC study patients’ characteristics at initial presentation with TTP-HUS.

	Mean or number (Percent)	Range
Age	49	16–85
Female	33 (69)	
Male	15 (31)	
Hemoglobin (g/dl)	9.0	2.5–13.4
Platelet count (x 10^9^/L)	4.6	5–307.000*
Serum creatinine (mg/dL	2.6	0.5–11.3
Idiopathic	35 (74)	
Pregnancy/Postpartum	2(4)	
Drug associated	3 (6)	
Autoimmune disorder (SLE and MG)	2 (4)	
Systemic infection	1 (2)	
Systemic Malignancy	4 (8)	
Renal transplant	1 (2)	

*Patient with platelet count of 307,000 x 10^9^/Lwas diagnosed with HUS due to Factor H mutation/ deficiency.

### Patients’ laboratory findings

As shown in Table 2, mean platelet count at presentation was 46 x 10^9^/ L (range 5 x 10^9^/ L—307 x 10^9^/ L). Mean hemoglobin was 9.0 g/dl (range 2.5–13.4 g/dl); mean creatinine concentration was 2.6 mg/dL (range 0.5–11.3 mg/dL). Mean CSS for our cohort was 4. Of note, baseline values and clinical severity score at our institution may not reflect the values/score at initial presentation because a few patients were transferred to our institution after steroid therapy was initiated at outside hospitals.

**Table 2:** Patient outcomes at UHCMC and comparison with relevant studies describing 30-day mortality and relapse.

Study	UHCMC2008–20125 years	Levandovsky, et al.1978-200224 years	Kim, et al.1998-200810 years	Lara, et al.1978-199820 years	George, et al.1989-200314.5 years	Roberts, et al.1984-19906 years
Total cases (total cases used for mortality rate calculation)	59 (59)	178 (167)^a^	52 (52)	126 (124)^c^	290 (214)^e^	14 (14)
Idiopathic cause (%)	75	72	27	71	39.3	43
Secondary cause (%)	25	28	73	29	60.7	57
Age	49*	49*	47*	49*	36** ^f^	45*
Gender (Female, %)	70	68	61.5	66	71	50
At presentation						
Hemoglobin (mg/dL)	9*	9.2*	7.6**	8.9 *	NS	9.8*
Serum creatinine (mg/dL)	2.6*	3.2*	2.5**	3,4**	1.2 ** ^f^	5.7 ^g^
Platelet count	46 x 10^9^/L*	49 x 10^9^/L*	30 x 10^9^/L**	44 x 10^9^/L*	11 x10^9^/L** ^f^	50 x 10^9^/L*
Clinical Severity Score	4*	4.4	NS	5	NS	NS
Complete response (%)	81.2	65	51.9	56	61^h^	NS
30days-Relapse rate (%)	18.6	18	NS	13	12	28.6
TTP-HUS related deaths 30-days (number and %)	4 (6.7%)	23 (14%) ^b^	18 (34.6%)	12 (10%) ^d^	38 (17%)	1(7%)
TPE as principal treatment (%)	100	96	100	97	100	100
Others principal treatment	N/A	FFP infusion and Staphylococcal protein A absorption column	N/A	FFP infusion, protein adsorption column therapy	N/A	N/A
Number of TPE procedures	12*	8**	5**	NS	20 ** ^f^	NS
TPE-Technique used	COBE Spectra	NS	COBE SpectraMFM (Plasauto)	Fenwal CS-3000 Blood cell separator,Haemonetics model V50, COBE Spectra	NS	NS

NS = Not specified

N/A = Not applicable

MFM = membrane filtration method

(a) 11 patients did not have death information and were excluded from the mortality rate calculation

(b) Total of 27 patients died but 4 patients died of causes unrelated to TTP-HUS and were excluded from the mortality rate calculation

(c) 2 patients’ charts were unavailable for review

(d) 1 patient died of an HIV-related infection and was excluded from the mortality rate calculation

(e) 76 patients had additional/alternative disorder and were excluded from the mortality rate calculation

(f) Detailed data from only 18 patients with severe ADAMTS13 deficiency were presented

(g) Calculated from mmol/L

(h) Defined as >150 x10^9^/L platelet count

* = mean

** = median

All patients started receiving and underwent TPE at our institution performed according to the manufacturer’s recommendations as well as our standard operating procedure. The daily procedures were done until achieving the target platelet level. Study patients received a mean of 12 TPEs (range 1–42); complete response was obtained in 81.2% of patients (N = 39/48) (platelet count > 100 x 10^9^/L); partial response in 8.3% of patients (N = 4/48) and 10.4% had no response (N = 5/48). Forty percent (N = 19/48) of the patients obtained platelet counts greater than 150 x 10^9^/L.

### Response relative to ADAMTS13 activity

In the overall cohort of 59 patients, those with severe ADAMTS13 deficiency (≤10%) required more TPE than those patients with enzyme deficiency >10% (mean 19.1 vs. 7.2). If the patients with ADAMTS13 deficiency are further subdivided into moderate deficiency (11%-40%), mild deficiency (41%-66%) and no deficiency (≥67%) there was no difference in the number of TPE in each subgroup (data not shown). For those patients who had a diagnosis of TTP-HUS and enzyme activity >10% the mean number of TPE received was 8.2. In our patient cohort, 8/59 (13.6%) patients had no ADAMTS13 measured at start or throughout TPE regimen. Of the 51/59 (86.4%) patients who had ADAMTS13 activity measured, 18/59 (30.5%) had severe metalloprotease deficiency (<10%); 16/59 (27.1%) patients had moderate to mild deficiency; and 17/59 (28.8%) patients had no deficiency in ADAMTS13 activity.

### Comparison with published literature

**Fig 2:**
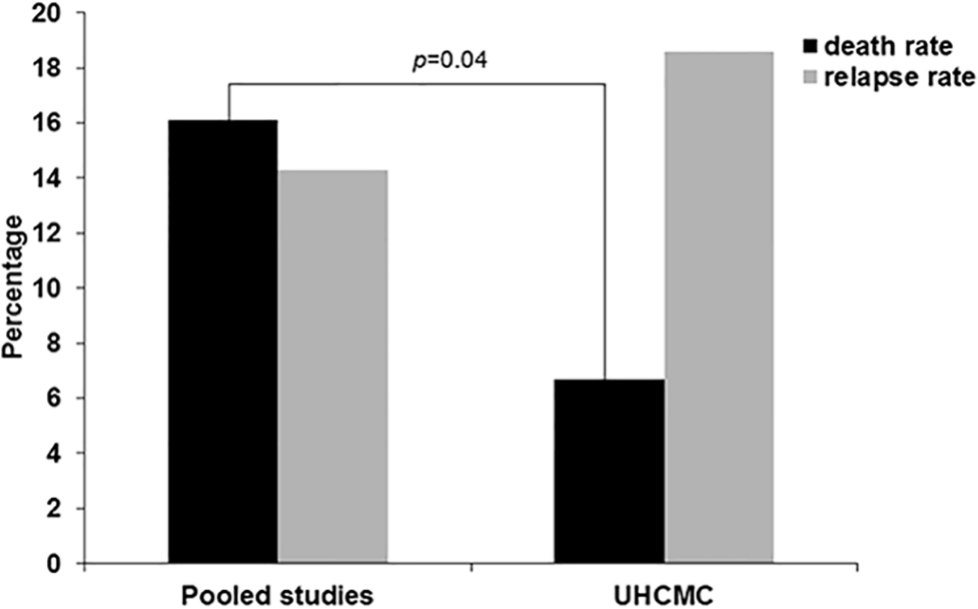
Thirty-day mortality and relapse in TTP-HUS. Thirty-day mortality attributed to TTP-HUS was 2% (1/48); however, overall mortality for patient cohort was 6.7% (4/59) (3 patients died of causes unrelated to TTP-HUS diagnosis (engraftment failure and graft vs. host disease, multi-organ failure due to sepsis and bacteremia, and heart failure due to worsening ejection fraction). Pooled 30-day mortality rate is 16.1% (92–571), *p* = 0.04. Relapse rate between our entire patient cohort (18.6%) and that of pooled data (14.3%).

We pooled the data of five studies that met the selection criteria (Fig 1 and Table 2). These five studies [12, 14, 16, 20, 21] described a total of 660 patients, 571 of which were used to calculate the mortality rate. Clinical Severity Score was derived only for two of the studies. Mean number of TPE are reported by 3/5 studies and practically all patients used in the statistical analysis received TPE. As shown in Fig 2, our overall mortality rate was significantly lower than the combined reported rate of all studies (4/59 (6.7%) vs. 92/571 (16%) respectively; *p* = 0.04). Our relapse rate, 11/59 (18.6%), was not significantly different from that which has been reported for the other studies used in our analysis (range 12–28%). However, when only those patients with TTP-HUS are taken into account the mortality rate (1/48) was more significant (*p* = 0.0053).

## Discussion

We have defined the clinical outcome of relapse and 30-day mortality in a population of 48 patients with a diagnosis of TTP-HUS. TPE was used as therapy for all patients whether the condition was idiopathic or secondary. Of interest, our 30-day mortality rate was significantly lower than what has been previously published but our relapse rate was similar to published reports. The marked difference in mortality rates may be difficult to dissect but in order to minimize confounding biases we compared our cohort findings to studies also reporting 30-day mortality rates and similar patient populations to ours since we were unable to perform a meta-analysis due to the small number of studies and limited raw data provided in these publications

An exhaustive search for treatment modalities/ interventions in published literature which included patients with either TTP or HUS found that patients with TTP tend to respond to TPE vs. patients with typical/ diarrheal HUS who respond best to supportive therapy that includes dialysis [22]. At our institution, all patients were given immunosuppression in the form of steroids which was given and discontinued at different intervals during TPE regimen as previously described [17]. This is similar to what has been described for a similar size patient cohort [23]. Therefore, the combination of immunosuppression and TPE may account for our improved survival. However, it did not have a role in decreasing relapse rates. In any case this is the therapeutic approach taken by most institutions (standard of care) and it is unlikely to explain our mortality differences with published reports. As a result, inherent differences in the patient cohorts which lead to marked differences in mortality may begin to explain our observations.

Studies which have grouped patients under TTP-HUS as a single clinical entity find that responses differ depending on the elements identified in the clinical presentation and patients’ gender [16]. Likewise, ABO blood group may be an independent risk factor for patients with TTP who have severe ADAMTS13 deficiency [24]. As a result, potential reasons for these reported differences include heterogeneity of patients diagnosed with TTP-HUS and serious underlying co-morbid conditions.

Since the majority of our patients had idiopathic TTP-HUS of which half had severe ADAMTS13 deficiency (TTP diagnosis), may make our better survival even more significant. Of interest, a recent report from our group showed that the response to TPE in patients with ADAMTS13 deficiency follows similar kinetics in platelet count recovery [25]. There is overlap between the two syndromes and the distinction between HUS and TTP based on routine laboratory and clinical presentation is at times challenging which has led to the unifying diagnosis of TTP-HUS to describe this patient population. For these reasons the term unifying both diagnoses into a single entity has been introduced [26]. However, if two potentially pathologically different disease entities were grouped, it could lead to differences in mortality rates and give creed to alternative suggestions for the markedly lower mortality rate that we observed in our patient cohort. In addition, unlike other studies included in our analysis, most of our patients had ADAMTS13 activity measured which was helpful in sub-stratifying our patient cohort. However, even subdividing patients according to ADAMTS13 activity still yielded a lower mortality rate than those prior reports.

The grouping of TTP and HUS into a unifying single pathologic entity may not represent a uniform way of studying, reporting and comparing these patients that is reproducible. A great deal of debate has placed by some investigators these two potential diseases under the same clinical umbrella as manifestations of a single disease continuum [13, 26, 27]. However, grouping these two clinical entities may oversimplify these two complex syndromic presentations that fails to recognize previously reported markedly different histopathologic findings in a large cohort of autopsied patients with either TTP or HUS which showed non-overlapping pathological signs, presentations that were distinct, and do not support the view that they represent a continuum of the same disease process [19]. Similarly, a meta-analysis looking at both clinical entities found that TTP patients respond to TPE leading to restoration of platelet count while HUS patients do not fully benefit from plasma therapy [28].

Molecularly, ADAMTS13 deficiency has to happen in the context of a “second hit” which is necessary for the clinical presentation to occur [29]. These could come in the form of both inhibitory and non-inhibitory antibodies to ADAMTS13 which could begin to explain why not all patients present with severe metalloprotease deficiency and that may also depend on the subjects’ genetic susceptibility as seen in different strains of ADAMTS13 knockout mice [29]. However, all ADAMTS13 deficient mice have shown a pro-thrombotic phenotype. The second hit could be in the form of an increase in vWF as shown in a TTP mouse model in which disease severity improved with the use of recombinant human ADAMTS13 [30]. Likely, disease dynamics of TTP and HUS may prove to be independent of each other with few if any intermediates mediating both pathologic presentations. In the case of TTP the correlation with ADAMTS13 deficiency may be stronger than the metalloprotease’s link to HUS [12]. Of note, though there is a wide variety of animal models that are used to determine the pathologic mediators behind TTP and HUS, these models have not yielded any shared molecule that is involved in the pathology of both [29]. More basic research is needed to dissect these possibilities further.

Additionally, promptness of TPE initiation is essential for patient survival [31] since in those whose diagnosis is delayed are more likely to die than those promptly treated [23]. Another possibility for our better outcomes is that at our institution TPE could be initiated earlier than others, based on clinical practice that patients with MAHA and thrombocytopenia of unknown etiology is suspected of TTP-HUS until proven otherwise. This is however unlikely. Along these lines, it has been proposed that adults with idiopathic disease characterized by unexplained thrombocytopenia, MAHA, normal international normalized ratio, partial thromboplastin time and D-dimer may have TTP-HUS [32]. However, this algorithm may be more based in caution than in actual evidence that these two disease entities are isoforms of a single syndromic presentation due to valid concerns that failure to timely treat a TTP diagnosis will lead to a potentially poor and complicated clinical outcome [32].

Differences in survival could also be due to lack of uniformity in plasma exchange practice, apheresis equipment used (COBE, membrane filtration method [16]; Staphylococcal protein A absorption column;[12] Fenwal CS-3000 Blood Separator and Haemonetics model V50 [14]; use of plasma infusion only [20]; and as adjunctive therapy [33]. Our institution has a TPE protocol, which is universally applied for all patients presenting with suspected TTP-HUS; additionally, as a tertiary referral center for others in our region, patients may begin adequate treatment prior to transfer or may not have survived their disease prior to transfer. These findings may begin to explain the observed discrepancies among studies.

In summary, our findings reinforce that early diagnosis; promptness of TPE initiation and use of TPE as standard treatment in the setting of TTP-HUS is essential to avoid the high 30-day mortality rate. Differences in survival may be due to lack of uniformity among the patient cohorts reported by us and others. Survival of TTP-HUS patients continues to improve; however, the marked differences in survivability need to be addressed with prospective studies that take into account the actual differences between these two disease presentations and negate the grouping of both under a single pathological entity.

## References

[1] Amorosi EL, Ultmann JE. Thrombotic thrombocytopenic purpura: report of 16 cases and review of the literature. Medicine (Baltimore). 1966;45:139–59.

[2] George JN. How I treat patients with thrombotic thrombocytopenic purpura: 2010. Blood. 2010;116(20):4060–9. Epub 2010/08/06. doi: blood-2010-07-271445 [pii]. 10.1182/blood-2010-07-271445 . 20686117

[3] Korkmaz S, Keklik M, Sivgin S, Yildirim R, Tombak A, Kaya ME, et al Therapeutic plasma exchange in patients with thrombotic thrombocytopenic purpura: a retrospective multicenter study. Transfus Apher Sci. 2013;48(3):353–8. Epub 2013/04/23. doi: S1473-0502(13)00101-8 [pii]. 10.1016/j.transci.2013.04.016 . 23602056

[4] Furlan M, Robles R, Galbusera M, Remuzzi G, Kyrle PA, Brenner B, et al von Willebrand factor-cleaving protease in thrombotic thrombocytopenic purpura and the hemolytic-uremic syndrome. N Engl J Med. 1998;339(22):1578–84. Epub 1998/11/26. 10.1056/NEJM199811263392202 . 9828245

[5] Tsai HM, Lian EC. Antibodies to von Willebrand factor-cleaving protease in acute thrombotic thrombocytopenic purpura. N Engl J Med. 1998;339(22):1585–94. Epub 1998/11/26. 10.1056/NEJM199811263392203 9828246 PMC3159001

[6] Remuzzi G, Galbusera M, Noris M, Canciani MT, Daina E, Bresin E, et al von Willebrand factor cleaving protease (ADAMTS13) is deficient in recurrent and familial thrombotic thrombocytopenic purpura and hemolytic uremic syndrome. Blood. 2002;100(3):778–85. Epub 2002/07/20. 10.1182/blood-2001-12-0166 . 12130486

[7] Vesely SK, George JN, Lammle B, Studt JD, Alberio L, El-Harake MA, et al ADAMTS13 activity in thrombotic thrombocytopenic purpura-hemolytic uremic syndrome: relation to presenting features and clinical outcomes in a prospective cohort of 142 patients. Blood. 2003;102(1):60–8. Epub 2003/03/15. doi: 10.1182/blood-2003-01-0193. 2003-01-0193 [pii]. . 12637323 10.1182/blood-2003-01-0193

[8] Amorosi EL, Karpatkin S. Antiplatelet treatment of thrombotic thrombocytopenic purpura. Ann Intern Med. 1977;86(1):102–6. Epub 1977/01/01. . 556919 10.7326/0003-4819-86-1-102

[9] Rock GA, Shumak KH, Buskard NA, Blanchette VS, Kelton JG, Nair RC, et al Comparison of plasma exchange with plasma infusion in the treatment of thrombotic thrombocytopenic purpura. Canadian Apheresis Study Group. N Engl J Med. 1991;325(6):393–7. Epub 1991/08/08. 10.1056/NEJM199108083250604 . 2062330

[10] Rock G, Shumak K, Kelton J, Blanchette VS, Buskard N, Nair R, et al Thrombotic thrombocytopenic purpura: outcome in 24 patients with renal impairment treated with plasma exchange. Canadian Apheresis Study Group. Transfusion. 1992;32(8):710–4. Epub 1992/10/01. . 1412677 10.1046/j.1537-2995.1992.32893032096.x

[11] Coppo P, Bengoufa D, Veyradier A, Wolf M, Bussel A, Millot GA, et al Severe ADAMTS13 deficiency in adult idiopathic thrombotic microangiopathies defines a subset of patients characterized by various autoimmune manifestations, lower platelet count, and mild renal involvement. Medicine (Baltimore). 2004;83(4):233–44. Epub 2004/07/03. doi: 00005792-200407000-00003 [pii]. . 15232311 10.1097/01.md.0000133622.03370.07

[12] Levandovsky M, Harvey D, Lara P, Wun T. Thrombotic thrombocytopenic purpura-hemolytic uremic syndrome (TTP-HUS): a 24-year clinical experience with 178 patients. J Hematol Oncol. 2008;1:23. Epub 2008/12/03. doi: 1756-8722-1-23 [pii]. 10.1186/1756-8722-1-23 19046460 PMC2613392

[13] George JN. How I treat patients with thrombotic thrombocytopenic purpura-hemolytic uremic syndrome. Blood. 2000;96(4):1223–9. Epub 2000/08/15. . 10942361

[14] Lara PN Jr., Coe TL, Zhou H, Fernando L, Holland PV, Wun T. Improved survival with plasma exchange in patients with thrombotic thrombocytopenic purpura-hemolytic uremic syndrome. Am J Med. 1999;107(6):573–9. Epub 2000/01/07. doi: S0002-9343(99)00286-7 [pii]. . 10625026 10.1016/s0002-9343(99)00286-7

[15] Kremer Hovinga JA, Vesely SK, Terrell DR, Lammle B, George JN. Survival and relapse in patients with thrombotic thrombocytopenic purpura. Blood. 2010;115(8):1500–11; quiz 662. Epub 2009/12/25. doi: blood-2009-09-243790 [pii]. 10.1182/blood-2009-09-243790 . 20032506

[16] Kim JW, Kim I, Oh KH, Yoon SS, Oh MD, Song YW, et al Therapeutic plasma exchange in patients with thrombotic thrombocytopenic purpura-hemolytic uremic syndrome: the 10-year experience of a single center. Hematology. 2011;16(2):73–9. Epub 2011/03/23. 10.1179/102453311X12902908411995 21418736

[17] Kier YE, Stempak LM, Maitta RW. Immature platelet fraction can help adjust therapy in refractory thrombotic microangiopathic hemolytic anemia cases. Transfus Apher Sci. 2013;49(3):644–6. Epub 2013/08/01. doi: S1473-0502(13)00232-2 [pii]. 10.1016/j.transci.2013.07.005 . 23899959

[18] Kokame K, Nobe Y, Kokubo Y, Okayama A, Miyata T. FRETS-VWF73, a first fluorogenic substrate for ADAMTS13 assay. Br J Haematol. 2005;129(1):93–100. Epub 2005/04/02. doi: BJH5420 [pii]. 10.1111/j.1365-2141.2005.05420.x . 15801961

[19] Hosler GA, Cusumano AM, Hutchins GM. Thrombotic thrombocytopenic purpura and hemolytic uremic syndrome are distinct pathologic entities. A review of 56 autopsy cases. Arch Pathol Lab Med. 2003;127(7):834–9. Epub 2003/06/26. 10.1043/1543-2165(2003)127<834:TTPAHU>2.0.CO;2 . 12823037

[20] George JN, Vesely SK, Terrell DR. The Oklahoma Thrombotic Thrombocytopenic Purpura-Hemolytic Uremic Syndrome (TTP-HUS) Registry: a community perspective of patients with clinically diagnosed TTP-HUS. Semin Hematol. 2004;41(1):60–7. Epub 2004/01/17. doi: S003719630300266X [pii]. . 14727260 10.1053/j.seminhematol.2003.10.001

[21] Roberts AW, Gillett EA, Fleming SJ. Hemolytic uremic syndrome/thrombotic thrombocytopenic purpura: outcome with plasma exchange. J Clin Apher. 1991;6(3):150–4. Epub 1991/01/01. . 1787131 10.1002/jca.2920060305

[22] Michael M, Elliott EJ, Ridley GF, Hodson EM, Craig JC. Interventions for haemolytic uraemic syndrome and thrombotic thrombocytopenic purpura. Cochrane Database Syst Rev. 2009;(1):CD003595 Epub 2009/01/23. 10.1002/14651858.CD003595.pub2 . 19160220 PMC7154575

[23] Dervenoulas J, Tsirigotis P, Bollas G, Pappa V, Xiros N, Economopoulos T, et al Thrombotic thrombocytopenic purpura/hemolytic uremic syndrome (TTP/HUS): treatment outcome, relapses, prognostic factors. A single-center experience of 48 cases. Ann Hematol. 2000;79(2):66–72. Epub 2000/03/31. . 10741917 10.1007/s002770050012

[24] Terrell DR, Motto DG, Kremer Hovinga JA, Lammle B, George JN, Vesely SK. Blood group O and black race are independent risk factors for thrombotic thrombocytopenic purpura associated with severe ADAMTS13 deficiency. Transfusion. 2011;51(10):2237–43. Epub 2011/04/08. 10.1111/j.1537-2995.2011.03125.x 21470236 PMC3151471

[25] Hong H, Xiao W, Stempak LM, Sandhaus LM, Maitta RW. Absolute immature platelet count dynamics in diagnosing and monitoring the clinical course of thrombotic thrombocytopenic purpura. Transfusion. 2014. Epub 2014/11/06. 10.1111/trf.12912 . 25370931

[26] Ruggenenti P, Remuzzi G. Thrombotic thrombocytopenic purpura and related disorders. Hematol Oncol Clin North Am. 1990;4(1):219–41. Epub 1990/02/01. . 2107174

[27] Remuzzi G. HUS and TTP: variable expression of a single entity. Kidney Int. 1987;32(2):292–308. Epub 1987/08/01. . 3309432 10.1038/ki.1987.206

[28] Garg AX, Suri RS, Barrowman N, Rehman F, Matsell D, Rosas-Arellano MP, et al Long-term renal prognosis of diarrhea-associated hemolytic uremic syndrome: a systematic review, meta-analysis, and meta-regression. JAMA. 2003;290(10):1360–70. Epub 2003/09/11. 10.1001/jama.290.10.1360290/10/1360 [pii]. . 12966129

[29] Vanhoorelbeke K, De Meyer SF. Animal models for thrombotic thrombocytopenic purpura. J Thromb Haemost. 2013;11 Suppl 1:2–10. Epub 2013/07/17. 10.1111/jth.12255 . 23809106

[30] Schiviz A, Wuersch K, Piskernik C, Dietrich B, Hoellriegl W, Rottensteiner H, et al A new mouse model mimicking thrombotic thrombocytopenic purpura: correction of symptoms by recombinant human ADAMTS13. Blood. 2012;119(25):6128–35. Epub 2012/04/25. doi: blood-2011-09-380535 [pii]. 10.1182/blood-2011-09-380535 . 22529289

[31] Colflesh CR, Agarwal R, Knochel JP. Timing of plasma exchange therapy for thrombotic thrombocytopenia purpura: a brief clinical observation. Am J Med Sci. 1996;311(4):167–8. Epub 1996/04/01. . 8602644 10.1097/00000441-199604000-00002

[32] Clark WF. Thrombotic microangiopathy: current knowledge and outcomes with plasma exchange. Semin Dial. 2012;25(2):214–9. Epub 2012/02/09. 10.1111/j.1525-139X.2011.01035.x . 22309967

[33] Forzley BR, Sontrop JM, Macnab JJ, Chen S, Clark WF. Treating TTP/HUS with plasma exchange: a single centre's 25-year experience. Br J Haematol. 2008;143(1):100–6. Epub 2008/08/12. doi: BJH7317 [pii]. 10.1111/j.1365-2141.2008.07317.x . 18691172

