# Correction: Re-Examination of 30-Day Survival and Relapse Rates in Patients with Thrombotic Thrombocytopenic Purpura-Hemolytic Uremic Syndrome

**DOI:** 10.1371/journal.pone.0131384

**Published:** 2015-06-22

**Authors:** Cassiana E. Bittencourt, Jennifer P. Ha, Robert W. Maitta

In Table 2, the calendar year and the number of years are merged in the subheadings. Please view the correct Table 2 below.

**Table 2:** Patient outcomes at UHCMC and comparison with relevant studies describing 30-day mortality and relapse.

2.Study	UHCMC 2008–2012 5 years	Levandovsky, et al. 1978–2002 24 years	Kim, et al. 1998–2008 10 years	Lara, et al. 1978–1998 20 years	George, et al 1989–2003 14.5 years	Roberts, et al. 1984–1990 6 years
Total cases (total cases used for mortality rate calculation)	59 (59)	178 (167)^a^	52 (52)	126 (124)^c^	290 (214)^e^	14 (14)
Idiopathic cause (%)	75	72	27	71	39.3	43
Secondary cause (%)	25	28	73	29	60.7	57
Age (mean* or median**)	49*	49*	47*	49*	36**^(f)^	45*
Gender (Female, %)	70	68	61.5	66	71	50%
At presentation						
Hemoglobin (mg/dL) mean* or median**	9*	9.2*	7.6**	8.9 *	NS	9.8*
Serum creatinine (mg/dL) mean* or median**	2.6*	3.2*	2.5**	3,4**	1.2 ** ^f^	5.7 ^g^
Platelet (mean* or median**)	46 x 10^9^/L*	49 x 10^9^/L*	30 x 10^9^/L**	44 x 10^9^/L*	11 x10^9^/L** ^f^	50 x 10^9^/L*
Clinical Severity Score (mean)	4*	4.4	NS	5	NS	NS
Complete response (%)	81.2	65	51.9	56	61^h^	NS
30days-Relapse rate (%)	18.6	18	NS	13	12	28.6
TTP-HUS related deaths 30-days (number and %)	4 (6.7%)	23 (14%) ^b^	18 (34.6%)	12 (10%) ^d^	38 (17%)	1(7%)
TPE as principal treatment (%)	100	96	100	97	100	100
Others principal treatment	N/A	FFP infusion and Staphylococcal protein A absorption column	N/A	FFP infusion Protein adsorption column therapy	N/A	N/A
Number of TPE procedures (mean* or median**)	12*	8**	5**	NS	20 ** ^f^	NS
TPE-Technique used	COBE Spectra	NS	COBE Spectra MFM (Plasauto)	Fenwal CS-3000 Blood Cell Separator Haemonetics model V50 Cobe Spectra	NS	NS
